# Risk factors for postoperative acute kidney injury in colorectal cancer: a systematic review and meta-analysis

**DOI:** 10.1007/s00384-025-04860-7

**Published:** 2025-03-18

**Authors:** Lumei Huang, Aifang Xiao, Yufeng Li

**Affiliations:** Department of Emergency, The Affiliated Taizhou People’s Hospital of Nanjing Medical University, Nanjing Medical University, Taizhou, Jiangsu Province China

**Keywords:** Colorectal cancer, Postoperative, Acute kidney injury, Risk factors, Meta-analysis

## Abstract

**Purpose:**

To thoroughly examine the risk factors that may predispose patients with colorectal cancer to postoperative acute kidney injury (AKI).

**Methods:**

To find relevant studies (from the beginning up to May 2024), two researchers searched PubMed, Web of Science, the Cochrane Library, and Embase databases. Two researchers evaluated the quality of the literature using the Newcastle–Ottawa Scale (NOS) and extracted data individually. Data analysis was performed using the Review Manager 5.4.

**Results:**

Our meta-analysis included 23 studies, encompassing a total of 167,904 patients. The identified risk factors for postoperative AKI in colorectal cancer patients were male sex, older age, body mass index (BMI) ≥ 25 kg/m^2^, hypertension, diabetes mellitus (DM), chronic kidney disease (CKD), hypoalbuminemia, emergency surgery, open surgery, prolonged operation time, American Society of Anesthesiologists (ASA) score ≥ 3, and intraoperative transfusion. In contrast, anemia and elevated creatinine levels did not emerge as significant risk factors for AKI in this population.

**Conclusion:**

To mitigate the incidence of postoperative AKI among these patients, healthcare professionals must proactively identify these risk factors and implement appropriate preventive measures.

## Introduction

Colorectal cancer (CRC) is the most common type of cancer in the world, with a high mortality rate [1]. The Global Cancer Statistics Report (GLOBOCAN) for 2020 indicates that CRC ranks as the third most commonly diagnosed cancer and the second leading cause of cancer-related deaths worldwide [2].

AKI, a life-threatening complication that can occur following surgery, is defined as a clinical condition caused by a rapid decline in kidney function, which can have multiple underlying causes [3]. Based on a comprehensive international survey, about 20% of patients had AKI after major surgeries [4]. This condition imposes a significant financial burden on the healthcare system worldwide [5]. AKI may contribute to a prolonged duration of hospitalization, escalate the expenses associated with hospitalization, and diminish the likelihood of survival after surgery [6]. What's more, the rate of late mortality remains rather high, regardless of whether renal function is restored before discharge [7].

Recently, academics have focused a great deal of attention on the risk factors for AKI in patients who have received colorectal cancer surgery [8]. AKI has been observed to occur in 3.8–19.5% of patients following colorectal cancer surgery [9]. The risk factors mentioned in each study differ, and few systematic analyses summarised the risk factors for AKI in patients with colorectal cancer [10]. Therefore, it is essential to identify the risk factors for AKI in patients who are undergoing colorectal cancer surgery and to apply appropriate intervention strategies to reduce the occurrence of AKI.

The purpose of this systematic review and meta-analysis was to pinpoint factors linked to postoperative AKI in individuals diagnosed with colorectal cancer. Additionally, it seeks to provide crucial insights for the early identification of patients at high risk and the development of effective management strategies.

## Methods

### Search strategy

The PRISMA guidelines were followed by every approach employed in this meta-analysis. We searched PubMed, Web of Science, Cochrane Library, and Embase databases for studies applicable to AKI in colorectal cancer patients (from inception to May 2024). We included the following keywords and medical subject headings: “colorectal cancer OR colorectal neoplasm OR colorectal OR colon cancer OR colon neoplasm OR rectal cancer OR rectal neoplasm” AND “acute kidney injury OR acute renal failure OR AKI OR ARF”.

### Inclusion and exclusion criteria

These were the inclusion criteria: (1) colorectal cancer patients diagnosed by preoperative colonoscopy and pathological biopsies; (2) patients aged ≥ 18 years who underwent colorectal surgery; (3) AKI defined clearly according to the Kidney Disease Improving Global Outcomes (KDIGO) criteria [11] and Acute Kidney Injury Network (AKIN) criteria [12]; (4) extractable data included odds ratios and 95% confidence intervals.

These were the exclusion criteria: (1) patients undergoing a second surgery for recurrent CRC; (2) patients with prior chronic renal failure; (3) duplicate or similar publications; and (4) conference abstracts, letters, case reports, and meta-analyses.

### Data extraction and quality assessment

Two researchers worked independently to collect the following data: first author, year, country, study type, type of surgery, incidence of AKI (n/total), risk factors, and AKI definition. They used the NOS independently to evaluate study quality. The NOS consists of three domains: population selection, comparison, and outcomes. The overall score ranges from 0 to 9, with scores of ≥ 7 indicating excellent quality, 5–6 indicating average quality, and < 4 indicating poor quality. A NOS score of 6 or above was deemed satisfactory for each study. If the two researchers were unable to reach an agreement, a third researcher joined the discussion to facilitate decision-making.

### Statistical analysis

The meta-analysis research was conducted with the Review Manager 5.4 software. We identified the factors that may predict AKI by combining the odds ratios (OR) and 95% confidence intervals (CI) obtained from each of the included studies. The assessment of heterogeneity was conducted using *I*^2^ and Cochran's Q test. When *P-value* > 0.1 and *I*^2^ < 50%, the fixed effects model was employed for the combination of the effect sizes. When *P-value* ≤ 0.1 and/or *I*^2^ ≥ 50%, a sensitivity analysis was performed to determine the source of the discrepancy. If the presence of heterogeneity persisted, we employed a random effects model to combine the effect sizes. A significance level of *P* < 0.05 was used for all comparisons.

## Results

### Study identification and selection

**Fig. 1:**
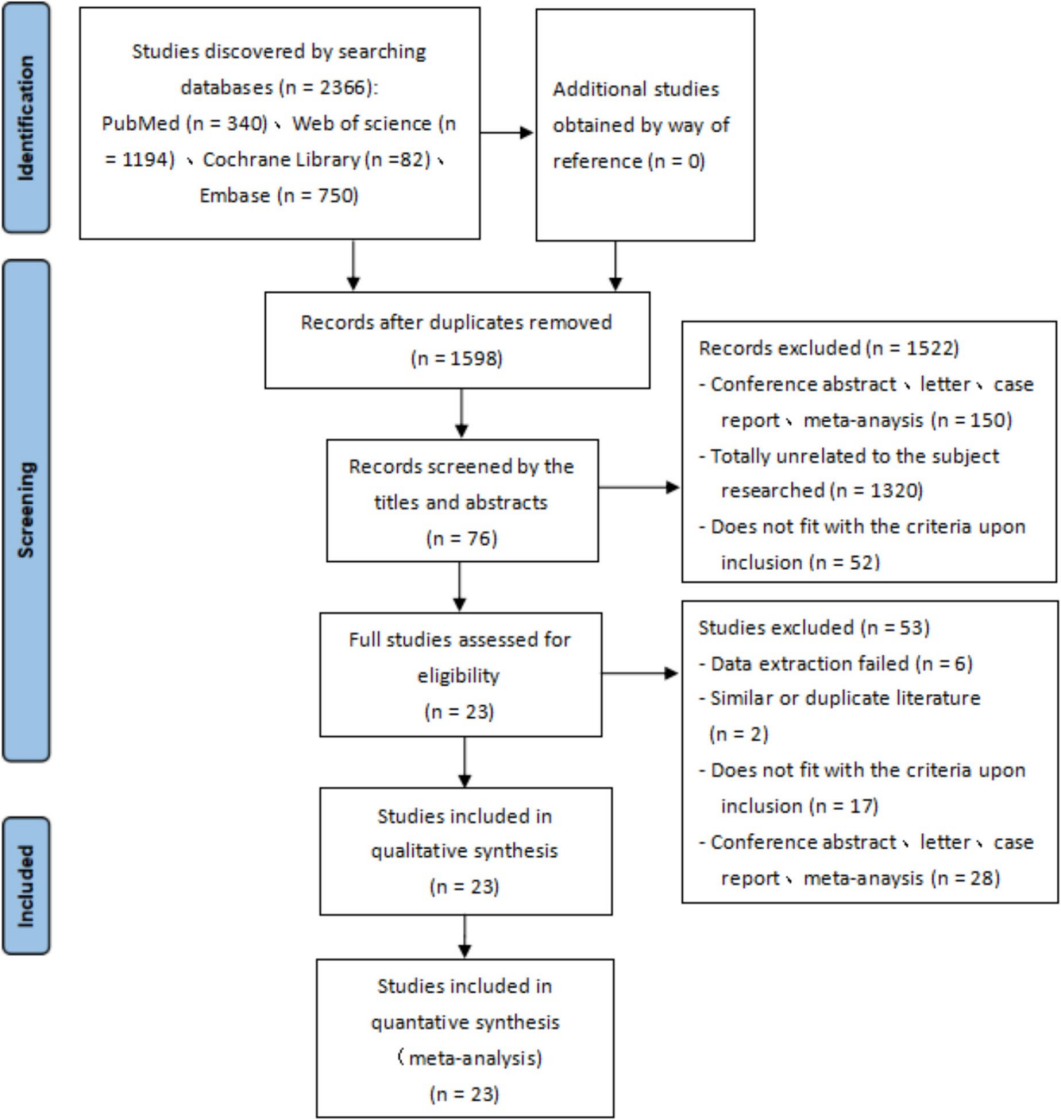
The flowchart for the relative studies selection procedure

Our initial research consisted of 2,366 references, from which we eliminated 768 duplicates. Following a review of titles and abstracts, we further excluded 1,522 references deemed irrelevant. The remaining 76 papers underwent a full-text examination, resulting in 23 being selected for both qualitative and quantitative synthesis. The methodology for this meta-analysis is illustrated in Fig. 1.

### Study characteristics and quality assessment

Among the 23 studies analyzed, 4 were case–control studies, 4 were prospective cohort studies, and the remaining 15 were retrospective cohort studies. The research includes a combined sample of 108,837 participants, among whom 15,829 experienced AKI. These studies were published between 2011 and 2023. The incidence of AKI in the selected articles ranged from 1.04% to 19.50%. Twenty-two of the studies achieved a score of 7 or higher, with only one study scoring 6, signifying the generally high quality of the research included. Table 1 presents the essential characteristics of the studies included, Table 2 presents the quality assessment.

**Table 1:** Essential characteristics of the studies included

Author	Year	Country	Study type	Type of surgery	Incidence of AKI	AKI	No-AKI	Risk factors	Definition
					(n/total)				
Causey [13]	2011	USA	Retrospective cohort	Colorectal surgery	11.80%	40	299	Intraoperative transfusion	KDIGO
Paquette [14]	2013	USA	Retrospective cohort	Colorectal surgery	7.46%	15	186	Older age	AKIN
Moghadamyeghaneh [15]	2014	UK	Retrospective cohort	Colorectal surgery	1.04%	290	27570	Male sex, older age, BMI ≥ 25 kg/m^2^, DM, emergency, open surgery	
Lim [16]	2016	Korea	Retrospective cohort	Rectal surgery	3.82%	11	277	DM	KDIGO
Bang [17]	2016	Korea	Retrospective cohort	Colorectal surgery	9.58%	414	3906	Older age, HTN, DM, emergency, hypoproteinemia	AKIN
Hassinger (1) [18]	2018	USA	Retrospective cohort	Colorectal surgery	12.67%	114	786	HTN, open surgery, prolonged operation time	KDIGO
Hassinger (2) [19]	2018	USA	Retrospective cohort	Colorectal surgery	11.51%	335	2575	Male sex, Older age, BMI ≥ 25 kg/m^2^, HTN, prolonged operation time	KDIGO
Grass [20]	2019	USA	Retrospective cohort	Colorectal surgery	2.54%	104	3993	DM, ASA score ≥ 3, prolonged operation time	KDIGO
Kadam [21]	2020	Australia	Retrospective cohort	Colorectal surgery	6.90%	52	702	ASA score ≥ 3	KDIGO
Wiener [22]	2020	USA	Retrospective cohort	Colorectal surgery	10.65%	112	940	ASA score ≥ 3	KDIGO
Shim [23]	2020	Korea	Retrospective cohort	Colorectal surgery	17.14%	79	382	Male sex, Older age, high BMI, hypoproteinemia	KDIGO
Essber [24]	2021	Israel	Case–control	Colorectal surgery	7.26%	268	3424	Open surgery	AKIN
Sim (1) [25]	2021	Korea	Retrospective cohort	Colorectal surgery	10.19%	361	3182	Male sex, BMI ≥ 25 kg/m^2^, HTN, DM, elevated creatinine levels	KDIGO
Sim (2) [26]	2021	Korea	Retrospective cohort	Colorectal surgery	9.05%	329	3308	HTN, DM, BMI ≥ 25 kg/m^2^, hypoproteinemia	KDIGO
Lumlertgul [27]	2021	UK	Prospective cohort	Colorectal surgery	13.48%	12	77	Intraoperative transfusion	KDIGO
Zorrilla-Vaca [28]	2021	USA	Prospective cohort	Colorectal surgery	7.69%	127	1525	Male sex, older age, ASA score ≥ 3, hypoalbuminemia, open surgery, CKD	KDIGO
Paek [29]	2021	Korea	Prospective cohort	Rectal surgery	12.06%	197	1436	Open surgery, intraoperative transfusion	KDIGO
Drakeford [30]	2022	Singapore	Case–control	Colorectal surgery	13.33%	74	481	Open surgery, elevated creatinine levels	KDIGO
Loria [9]	2022	USA	Prospective cohort	Colorectal surgery	19.50%	51	262	Elevated creatinine levels	KDIGO
Li [31]	2023	China	Retrospective cohort	Colorectal surgery	7.71%	30	359	HTN, anemia	KDIGO
Thanh [32]	2023	Vietnam	Case–control	Colorectal surgery	14.35%	31	185	DM, anemia	KDIGO
Omar [33]	2023	France	Retrospective cohort	Rectal surgery	18.05%	50	227	Older age, ASA score ≥ 3	AKIN
Andresen [8]	2023	UK	Case–control	Colorectal surgery	14.34%	15,612	93,225	Older age, CKD	KDIGO

Abbreviations:* AKI, *Acute Kidney Injury;* HTN*, Hypertension; *BMI*, Body Mass Index; *DM*, Diabetes Mellitus; *CKD*, Chronic Kidney Disease; *ASA*, American Society of Anesthesiologists

**Table 2:** The quality assessment of the included studies

Study	Selection				Comparability	Outcome			Total score
Causey [13]	1	1	0	1	1	0	1	1	6
Paquette [14]	1	1	1	1	1	0	1	1	7
Moghadamyeghaneh [15]	1	1	0	1	1	1	1	1	7
Lim [16]	1	1	1	1	1	1	1	1	8
Bang [17]	1	1	1	1	1	1	1	1	8
Hassinger (1) [18]	1	1	0	1	1	1	1	1	7
Hassinger (2) [19]	1	1	0	1	1	1	1	1	7
Grass [20]	1	1	1	1	1	1	1	1	8
Kadam [21]	1	1	1	1	1	1	1	1	8
Wiener [22]	1	1	1	1	1	1	1	1	8
Shim [23]	1	1	0	1	1	1	1	1	7
Essber [24]	1	1	1	1	1	0	1	1	7
Sim (1) [25]	1	1	1	1	1	1	1	1	8
Sim (2) [26]	1	1	0	1	1	1	1	1	7
Lumlertgul [27]	1	1	1	1	1	1	1	1	8
Zorrilla-Vaca [28]	1	1	1	1	1	1	1	1	8
Paek [29]	1	1	1	1	1	1	1	1	8
Drakeford [30]	1	1	1	1	1	1	1	1	8
Loria [9]	1	1	1	1	1	1	1	1	8
Li [31]	1	1	1	1	2	1	1	1	9
Thanh [32]	1	1	1	1	1	1	1	1	8
Omar [33]	1	1	1	1	1	0	1	1	7
Andresen [8]	1	1	1	1	2	1	1	1	9

### Risk factors for AKI

#### Male sex

**Fig. 2:**
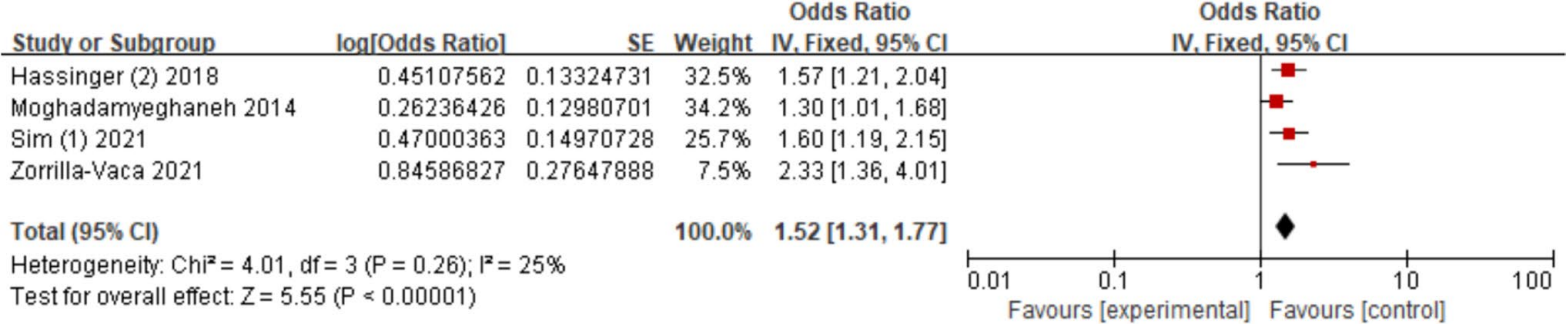
Forest plot for male sex

Five studies [15, 19, 23, 25, 28] assessed the relationship between male sex and AKI. Through conducting a sensitivity analysis, we removed the research [23], resulting in a decrease in the *I*^2^ value to 25%. The findings demonstrated that male patients had a greater AKI risk than female patients (OR = 1.52; 95% CI, 1.31–1.77, *P* < 0.00001). Figure 2.

#### Older age

**Fig. 3:**
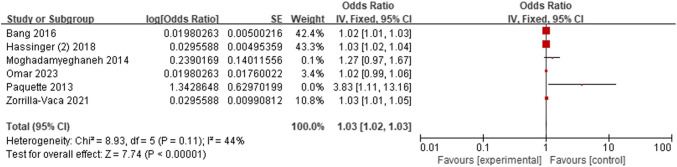
Forest plot for older age

Seven studies [14, 15, 17, 19, 23, 28, 33] evaluated the relationship between older age and AKI. Through conducting a sensitivity analysis, we removed the research [23], resulting in a decrease in the *I*^2^ value to 44%. The findings demonstrated that older age increased AKI risk (OR = 1.03; 95% CI, 1.02–1.03, *P* < 0.00001). Figure 3. Andresen et al. [8] estimated that older age was a risk factor for AKI (*P* < 0.05), although the results were not comparable due to continuous values.

#### BMI ≥ 25 kg/m^2^

**Fig. 4:**
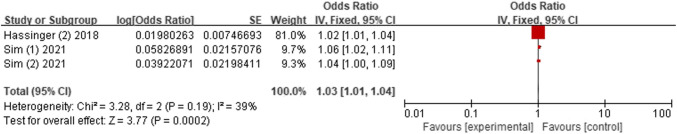
Forest plot for BMI ≥ 25 kg/m^2^

Four studies [15, 19, 25, 26] assessed the relationship between BMI ≥ 25 kg/m^2^ and AKI. Through conducting a sensitivity analysis, we removed the research [15], resulting in a decrease in the *I*^2^ value to 39%. The findings demonstrated that BMI ≥ 25 kg/m^2^ increased AKI risk (OR = 1.03; 95% CI, 1.01–1.04, *P* = 0.0002). Figure 4. In addition, Shim et al. [23] estimated that higher BMI was a risk factor for AKI (*P* < 0.05), but the continuous BMI value prevented combined analysis.

#### Hypertension

**Fig. 5:**
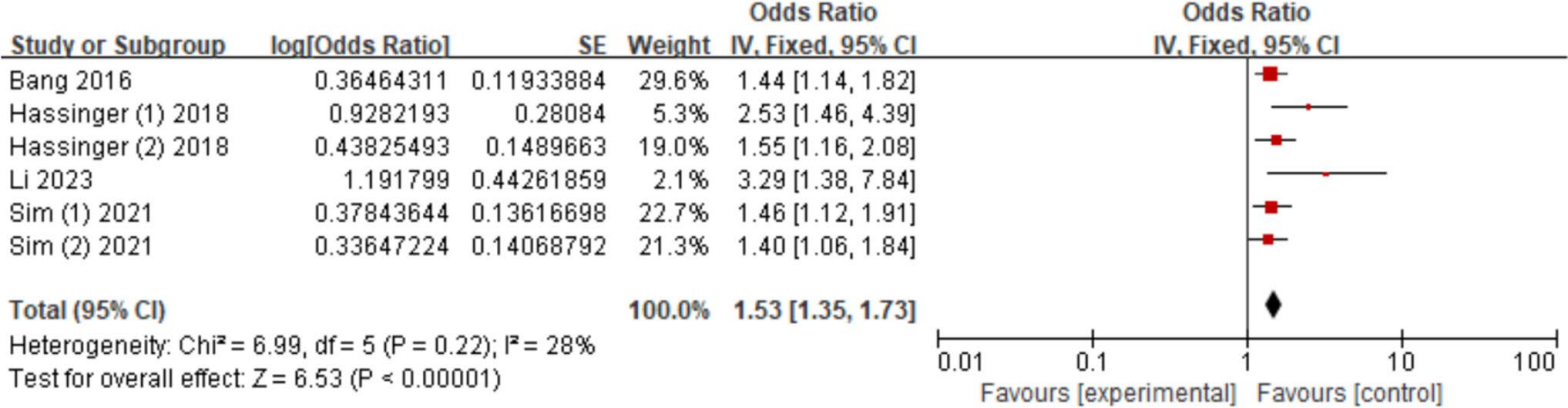
Forest plot for hypertension

Six studies [17–19, 25, 26, 31] assessed the relationship between hypertension and AKI. The findings demonstrated that hypertension increased AKI risk (OR = 1.53; 95% CI, 1.35–1.73, *P* < 0.00001). Figure 5.

#### Diabetes mellitus

**Fig. 6:**
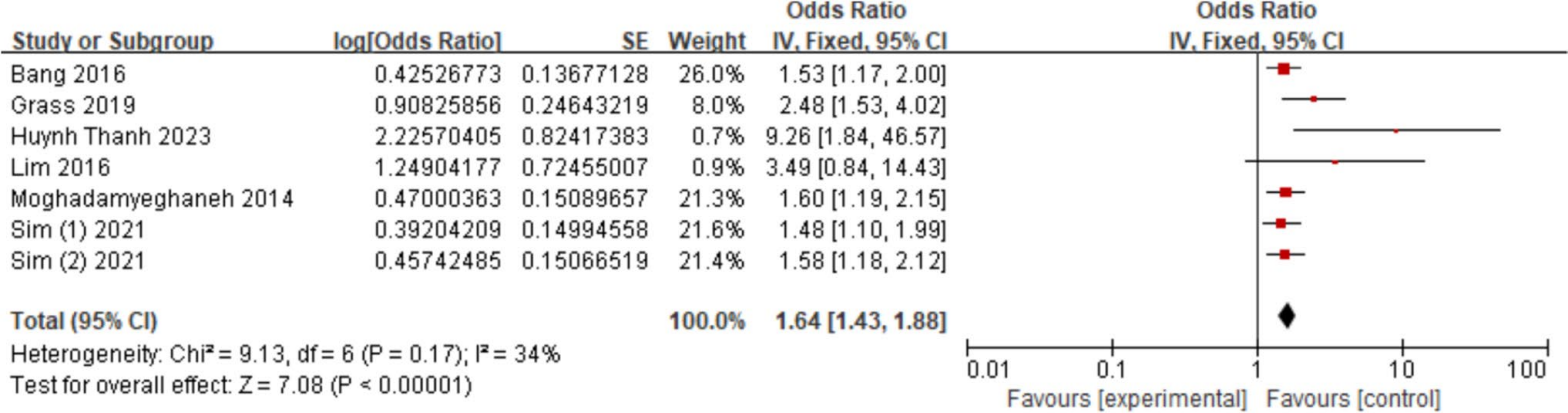
Forest plot for diabetes melltius

Seven studies [15–17, 20, 25, 26, 32] assessed the relationship between diabetes melltius and AKI. The findings demonstrated that the diabetes melltius increased AKI risk (OR = 1.64; 95% CI, 1.43–1.88, *P* < 0.00001). Figure 6.

#### Chronic kidney diseases

**Fig. 7:**
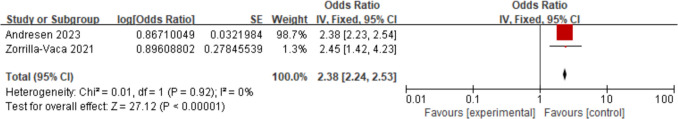
Forest plot for chronic kidney diseases

Two studies [8, 28] assessed the relationship between chronic kidney diseases and AKI. The findings demonstrated that chronic kidney diseases increased AKI risk (OR = 2.38; 95% CI, 2.24–2.53, *P* < 0.00001). Figure 7.

#### Anemia

**Fig. 8:**
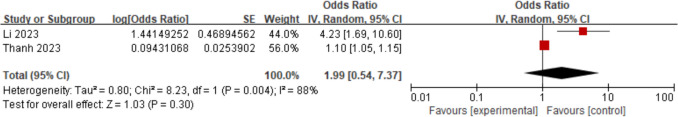
Forest plot for anemia

Two studies [31, 32] assessed the relationship between anemia and AKI. The findings demonstrated that anemia was not a risk factor for AKI (OR = 1.99; 95% CI, 0.54–7.37, *P* = 0.30). Figure 8.

#### Hypoproteinemia

**Fig. 9:**
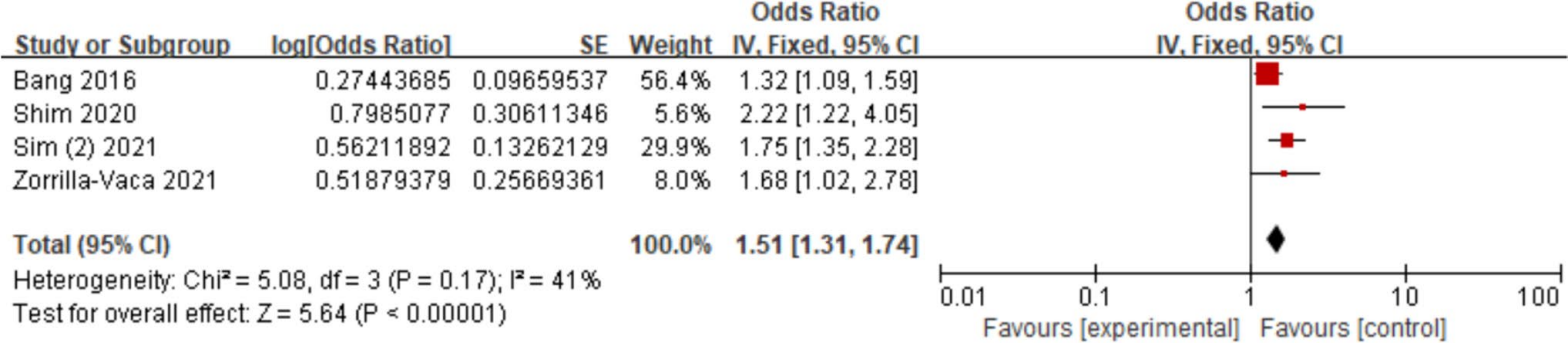
Forest plot for hypoproteinemia

Four studies [17, 23, 26, 28] assessed the relationship between hypoproteinemia and AKI. The findings demonstrated that hypoproteinemia increased AKI risk (OR = 1.51; 95% CI, 1.31–1.74, *P* < 0.00001). Figure 9.

#### Elevated creatinine levels

**Fig. 10:**
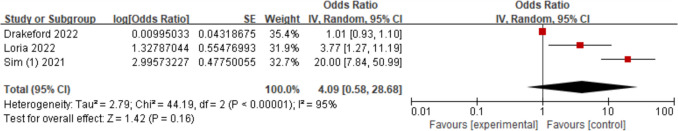
Forest plot for elevated creatinine levels

Three studies [9, 25, 30] assessed the relationship between elevated creatinine levels and AKI. We used a random effect model to combine effect sizes. The findings demonstrated that elevated creatinine levels did not predict AKI (OR = 4.09; 95% CI, 0.58–28.68, *P* = 0.16). Figure 10.

#### Emergency surgery

**Fig. 11:**
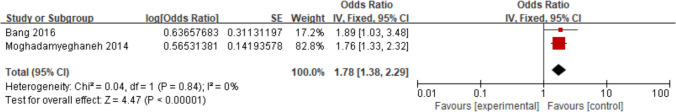
Forest plot for emergency surgery

Two studies [15, 17] assessed the relationship between emergency surgery and AKI. The findings demonstrated that AKI was more likely to occur after emergency surgery (OR = 1.78; 95% CI, 1.38–2.29, *P* < 0.00001). Figure 11.

#### Open surgery

**Fig. 12:**
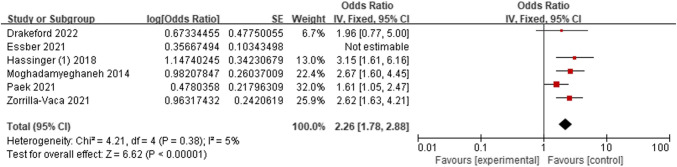
Forest plot for open surgery

Six studies [15, 18, 24, 28–30] assessed the relationship between open surgery and AKI. Through conducting a sensitivity analysis, we removed the research [24], resulting in a decrease in the *I*^2^ value to 5%. The findings demonstrated that open surgery increased the risk of AKI compared to laparoscopic surgery (OR = 2.26; 95% CI, 1.78–2.88, *P* < 0.0001). Figure 12.

#### Prolonged operation time

**Fig. 13:**
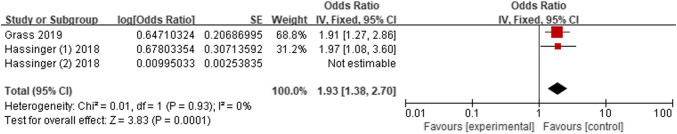
Forest plot for prolonged operation time

Three studies [18–20] assessed the relationship between prolonged operation time and AKI. Through conducting a sensitivity analysis, we removed the research [19], resulting in a decrease in the *I*^2^ value to 0%. The findings demonstrated that the prolonged operation time increased AKI risk (OR = 1.93; 95% CI, 1.38–2.70, *P* = 0.0001). Figure 13.

#### ASA score ≥ 3

**Fig. 14:**
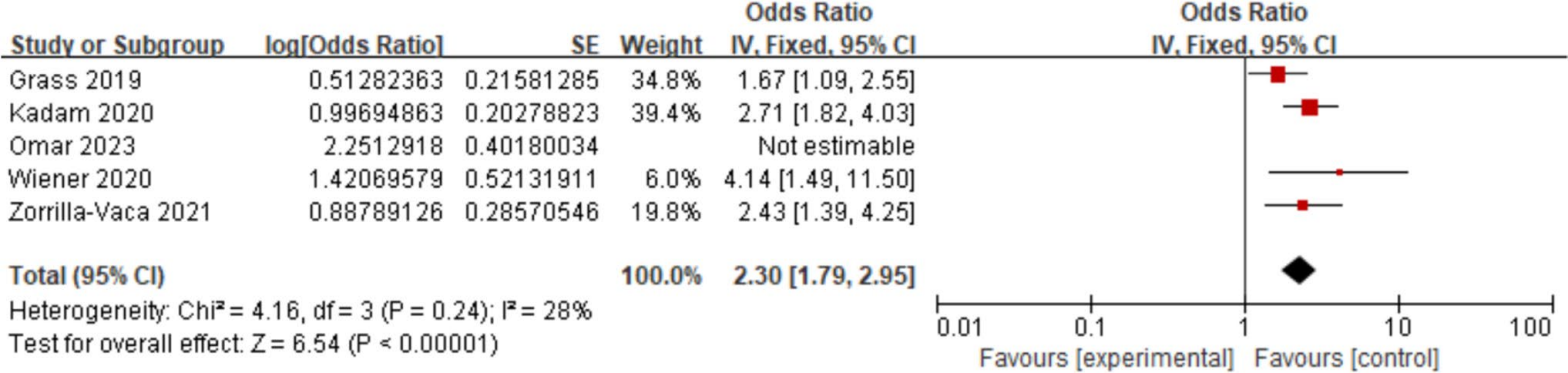
Forest plot for ASA score ≥ 3

Five studies [20–22, 28, 33] assessed the relationship between an ASA score ≥ 3 and AKI. Through conducting a sensitivity analysis, we removed the study [28], resulting in a decrease in the *I*^2^ value to 28%. The findings demonstrated that patients with an ASA score ≥ 3 faced a heightened risk of AKI (OR = 2.30; 95% CI, 1.79–2.95, *P* < 0.00001). Figure 14.

#### Intraoperative transfusion

**Fig. 15:**
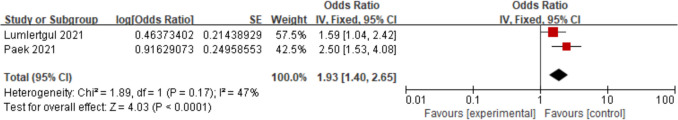
Forest plot for intraoperative transfusion

Three studies [13, 27, 29] assessed the relationship between intraoperative transfusion and AKI. Through conducting a sensitivity analysis, we removed study [13], resulting in a decrease in the *I*^2^ value to 47%. The findings demonstrated that receiving a blood transfusion after surgery was associated with an increased risk of AKI (OR = 1.93; 95% CI, 1.40–2.65, *P* < 0.0001). Figure 15.

## Discussion

The incidence and risk factors of AKI in individuals with colorectal cancer remain a topic of active debate. This study is the first meta-analysis exploring the risk factors associated with AKI in patients who have undergone surgery for colorectal cancer. Risk factors for AKI include male sex, older age, BMI ≥ 25 kg/m^2^, hypertension, DM, CKD, hypoalbuminemia, emergency surgery, open surgery, prolonged operation time, ASA score ≥ 3, and intraoperative transfusion are risk factors for AKI in cancer following colorectal surgery. Among them, male sex, older age, BMI ≥ 25 kg/m^2^ belonged to general demographic factors, hypertension, DM, CKD, hypoalbuminemia belonged to disease-related factors, emergency surgery, open surgery, prolonged operation time, ASA score ≥ 3, and intraoperative transfusion belonged to surgery-related risk factors. The findings could facilitate early identification of colorectal cancer patients at risk for postoperative AKI, enabling prompt intervention to reduce AKI occurrence and improve patient prognosis and quality of life.

### General demographic factors

Males might be more susceptible to AKI following surgery, potentially due to lower estrogen levels; indeed female estrogen appear to offer a protective effect on the kidneys [34]. The elderly are susceptible to AKI due to due to alterations in hemodynamics, disruptions in water and electrolyte balance, acid–base imbalances, and reduced renal physiological reserve function and operational tolerance [35]. Moreover, elderly patients often have preexisting conditions like hypertension and diabetes, which increase the strain on the kidneys [36]. Patients with a high BMI are prone to adipose tissue buildup in the body, leading to an augmented strain on the kidneys and ultimately culminating in renal disease [37]. Mounting evidence indicates a robust correlation between AKI in patients and the development of chronic kidney disease [38, 39].

### Disease-related factors

Persistent hypertension may result in physiological anomalies in the kidney, which can rapidly lead to a decline in renal function following surgical procedures and periods of stress [40]. Prolonged use of antihypertensive medications, such as ACE inhibitors or angiotensin receptor blockers (ARBs), in individuals with hypertension reduces the kidneys' ability to tolerate the toxic effects of some pharmaceuticals, thereby increasing the likelihood of postoperative AKI [41]. Recent studies have demonstrated that intraoperative blood pressure fluctuation is strongly connected to postoperative AKI, suggesting potential directions for future studies [42, 43]. Hyperglycemia disrupts the innate regulation of renal blood flow, reduces the patient's tolerance to reduced blood supply during surgery, and increases the risk of AKI post-operation. Concurrently, elevated blood glucose levels can exacerbate inflammation, lead to the accumulation of oxidative products, impair vasodilation, and reduce renal blood flow. Hapca et al. [44] illustrated that diabetic patients, in the absence of CKD, are nearly five times more likely to develop AKI than those without diabetes. Therefore, it is crucial to assess patients' blood glucose levels prior to colorectal surgery and to enhance the management of their blood sugar. Research has demonstrated that albumin can alleviate the harmful effects of medications on the kidneys, maintain the functionality of glomerular filtration, and thereby improve microcirculation [45]. However, individuals suffering from hypoalbuminemia may experience compromised integrity of glycolysis, leading to the dissipation of osmotic pressure gradients and diminished barrier function [46]. Consequently, patients with preoperative hypoalbuminemia require significant enhancement of their nutritional intake.

### Surgery-related risk factors

Emergency surgery is a strong risk factor for AKI. Sevoflurane anesthesia is commonly administered to patients in emergency situations. Bang et al. [17] identified sevoflurane as a predictor of AKI. Among colorectal procedures, open surgery carries the highest risk of postoperative AKI. Essber et al. [24] revealed that laparoscopic surgery significantly reduces the risk of AKI, with patients undergoing this procedure having a 30% lower chance of developing AKI than those undergoing open surgery (OR = 0.7, *p* < 0.05). Furthermore, various studies [26, 29] have shown that individuals undergoing laparoscopic surgery experience fewer complications and a higher survival rate than those undergoing open surgery. A prolonged duration of surgery may indicate a complex medical condition and a challenging surgical procedure, potentially leading to kidney injury of varying degrees, either directly or indirectly [47]. During a blood transfusion, patients receive a significant volume of plasma and red blood cells that need processing and excretion by the kidneys, potentially leading to increased renal workload. Cao et al. [48] found that individuals who maintained hemoglobin levels above 9 g/dL via red blood cell transfusions had a higher risk of AKI.

The meta-analysis indicated that neither anemia nor postoperative creatinine levels were predictors of AKI. Given that only two studies were included, this finding may be affected by the limited available literature. There is an urgent need for additional high-quality research specifically focused on anemia and postoperative creatinine levels in patients with colorectal cancer.

### Limitations

When interpreting the results of this meta-analysis, several limitations should be considered. First, the majority of the included studies were retrospective, which may introduce inherent biases into the data. Second, there was a lack of uniformity in AKI definitions across the studies, potentially affecting outcomes and contributing to variability. Third, it is important to note that some pooled results in this meta-analysis were derived from a limited number of studies, which may impact their reliability. Finally, certain risk factors, such as postoperative bowel ileus, sevoflurane, and diuretic use, were limited by study availability, restricting the scope for comprehensive analysis. To better understand these associated factors and reduce the incidence of AKI in colorectal cancer patients, we advocate for conducting large-scale, multicenter, prospective studies in the near future.

## Conclusion

Our research identified several risk factors for AKI in patients with colorectal cancer, including male sex, older age, BMI ≥ 25 kg/m^2^, hypertension, DM, CKD, hypoalbuminemia, emergency surgery, open surgery, prolonged operation time, ASA score ≥ 3, and intraoperative transfusion. Conversely, anemia and postoperative creatinine levels were not associated with an increased risk of AKI in this population. We anticipate that our findings will provide valuable insights to guide future therapeutic strategies aimed at reducing the incidence of AKI and improving patients' overall quality of life.

## Data Availability

No datasets were generated or analysed during the current study.
